# Case of Missing Plastic: Foreign Body Bronchiectasis

**DOI:** 10.7759/cureus.2974

**Published:** 2018-07-12

**Authors:** Saketh Palasamudram Shekar, Pablo Bajarano, Anas Hadeh, Edward Rojas, Samantha R Gillenwater, Edward Savage, Jinesh PpP Mehta

**Affiliations:** 1 Pulmonary and Critical Care, Cleveland Clinic Florida, Weston, USA; 2 Pathology, Cleveland Clinic Florida, Weston, USA; 3 Internal Medicine, Cleveland Clinic Florida, Weston, USA; 4 Cardiothoracic Surgery, Cleveland Clinic Florida, Weston, USA

**Keywords:** non cystic fibrosis bronchiectasis, surgical pathology, foreign body

## Abstract

Bronchiectasis is a well-known entity where the airways abnormally dilate losing their natural function. Most common causes of non-cytic fibrosis bronchiectasis in the middle age group include secondary immunodeficiency, aspiration, and allergic bronchopulmonary aspergillosis (ABPA). Obstructive foreign body is an uncommon cause of bronchiectasis and is often a missed diagnosis in a localized disease. Foreign bodies can be missed making the diagnosis and treatment more challenging and hence foreign body bronchiectasis should be considered in patients presenting with focal disease. Here we describe a patient with a retained foreign body that was discovered post lobectomy during gross pathological examination of the specimen with no significant aspiration history, non-diagnostic imaging of the chest and negative bronchoscopy.

## Introduction

Bronchiectasis is a clinical entity where the airways are permanently and abnormally dilated as a consequence of an insult [1]. Bronchiectatic airways lose their function including mucociliary clearance which results in bacterial overgrowth and repeated infections [2]. Many insults have been identified to cause the damage of the airways. Most common causes in middle-aged patients are metastatic malignancy, secondary immunodeficiency syndromes, allergic bronchopulmonary aspergillosis (ABPA), chronic aspiration and retained foreign body. Frequently the cause of bronchiectasis is unknown. Here we present a case of focal bronchiectasis with bronchiectasis severity index (BSI) of 15, indicating high one-year mortality [3] in whom the etiology was discovered to be a retained foreign body post lobectomy in a pathology specimen.

## Case presentation

Fifty-nine-year-old woman presented to a pulmonary outpatient department for management of recurrent pneumonias due to bronchiectasis diagnosed two years ago. She was found to have on an average of four to five episodes of lower respiratory tract infections (LRTI) per year during the same period. She was apparently well until about two years ago when she started developing LRTI which was initially treated with various antibiotics including fluoroquinolones, beta-lactam antibiotics and macrolides at different walk-in clinics. Chest X-rays obtained before the presentation were normal. Computed tomography (CT) of the chest which was obtained at our hospital revealed right lower lobe focal cylindrical bronchiectasis. This was presumed to be post-infectious based on LRTI history. Frequency of LRTI increased from an average of two episodes in six months to three to four episodes in six months. Repeat sputum cultures grew pseudomonas aeruginosa every time. She was managed symptomatically with airways mucus clearance and fluoroquinolones as needed.

**Figure 1:**
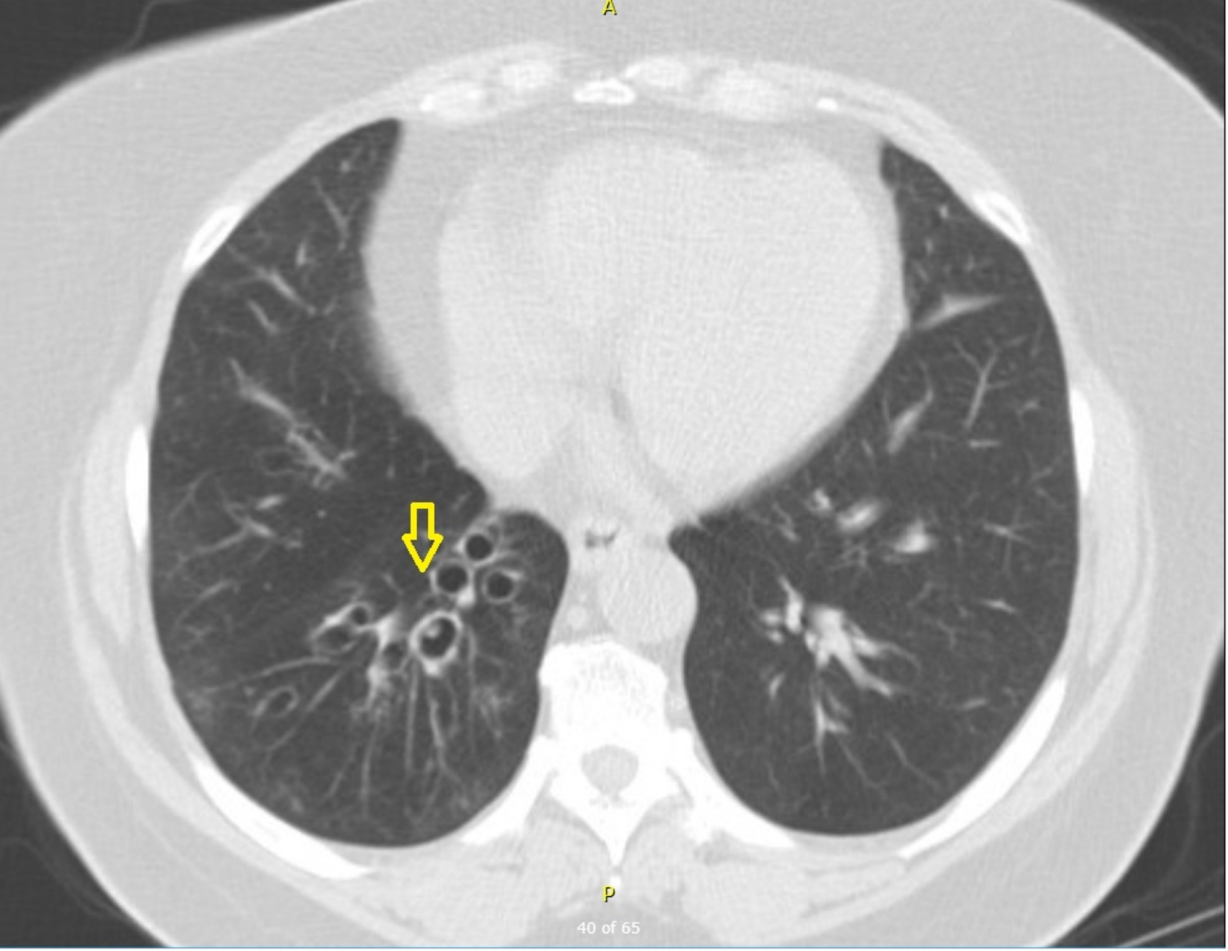
Computed tomography of the chest. Axial view of computed tomography of the chest at the level of lower lobes indicating bronchiectatic changes of the airway (arrow).

**Figure 2:**
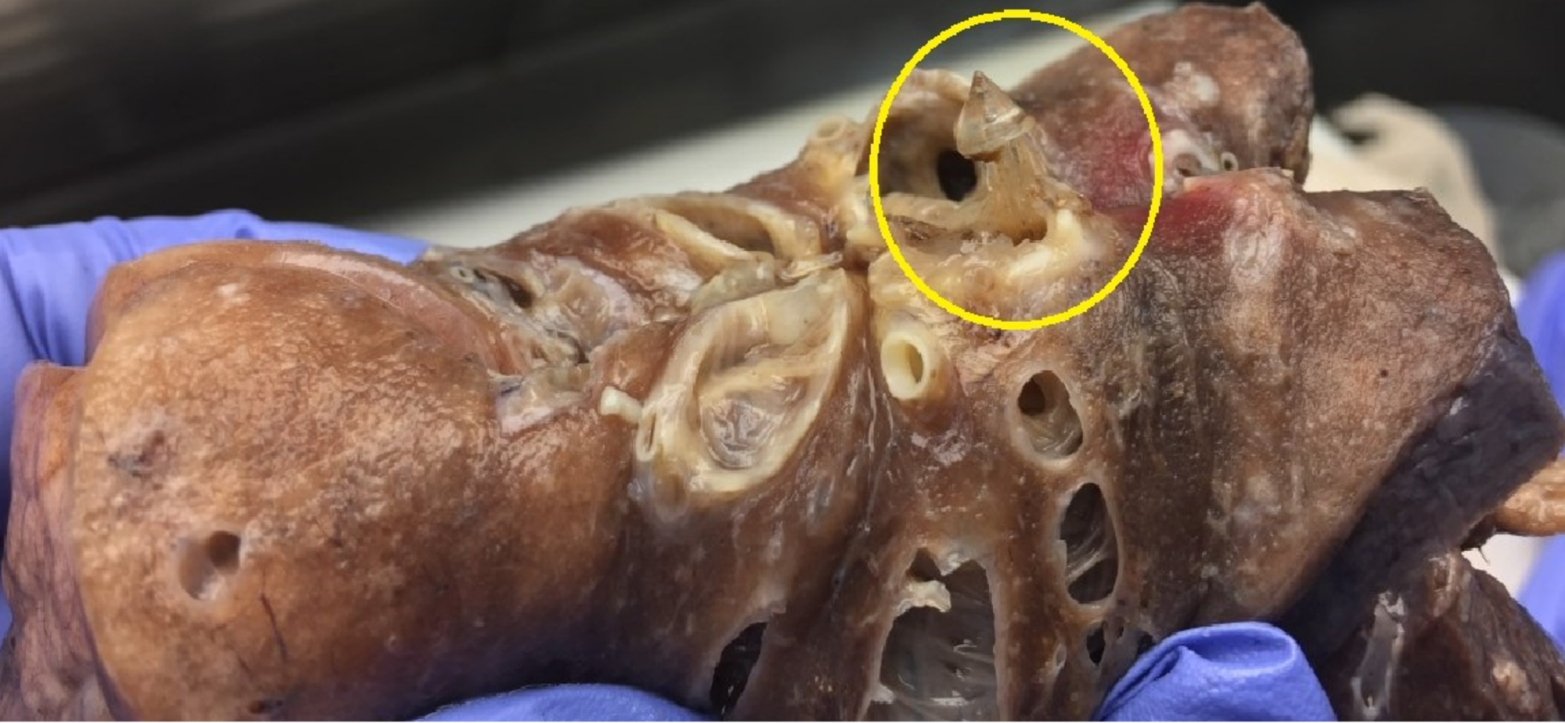
Pathology gross specimen. Gross specimen of the resected right lower lobe showing the non-surgical foreign body (circle).

**Figure 3:**
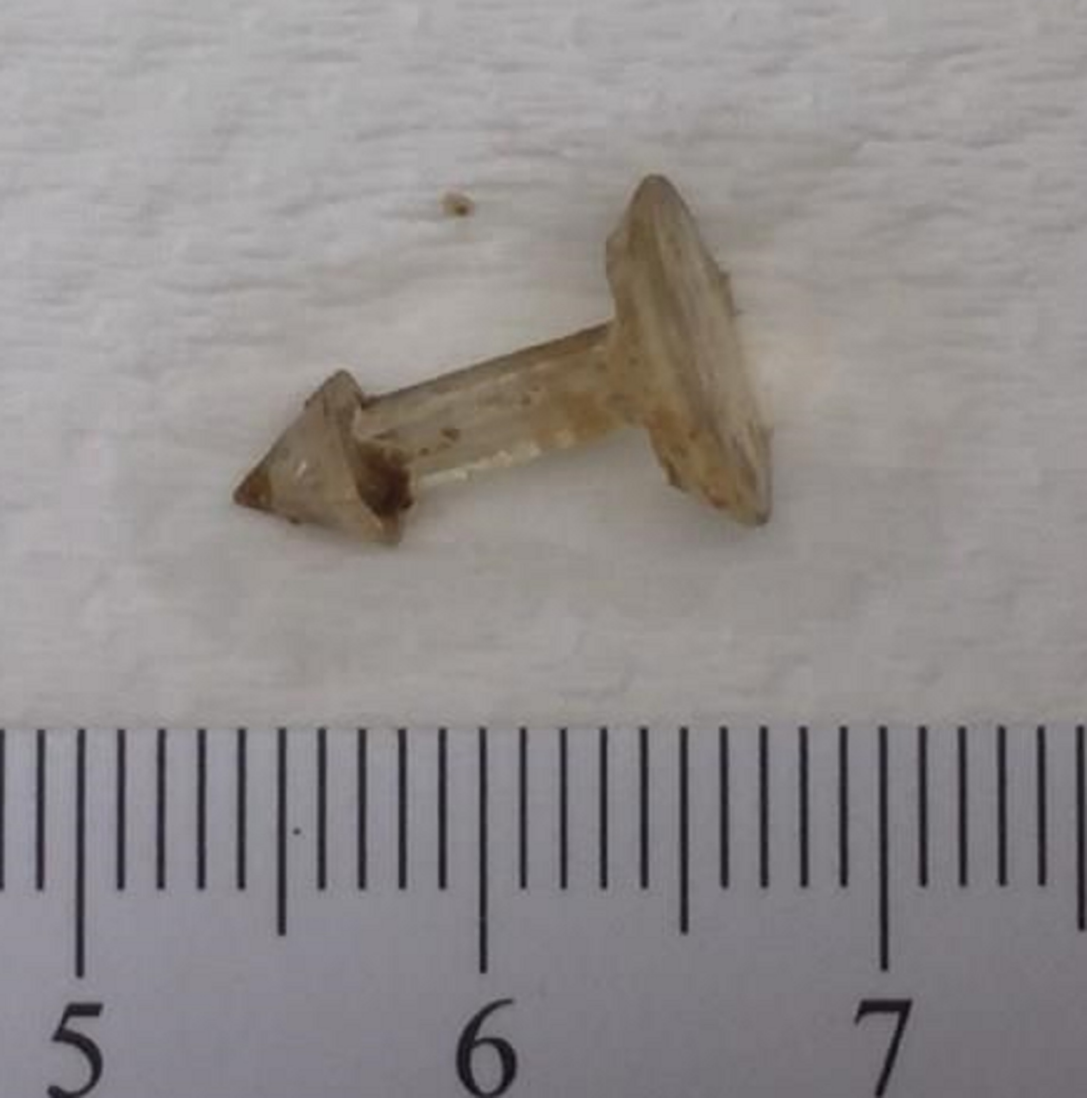
The retained foreign body (scale in centimeters). Retrieved foreign body during pathological examination of the gross specimen.

History for pulmonary childhood infections, immunodeficiency, severe allergies and other risk factors for bronchiectasis was negative. Serum immunoglobulin G subgroups and immunoglobulin E levels were normal arguing against immunodeficiency and ABPA. Upon closer review of the computed tomography of the chest, a possible endobronchial lesion was noticed resembling a polyp of about half a centimeter in length (Figure 1) at the right lower lobe posterior segment. Flexible bronchoscopy was performed revealing thick yellow-green secretions originating from the right lower lobe with an endobronchial lesion in the posterior segment at the same level as the CT scan abnormality. Cultures from the bronchoalveolar once again grew pseudomonas aeruginosa and pathology from the biopsy of the endobronchial mass revealed acute inflammation with predominant neutrophils which we related to ongoing infection. Over the next six months, the patient had three hospitalizations due to LRTI. Department of cardiothoracic surgery was consulted and the patient underwent a video-assisted thoracoscopic surgery and a right lower lobectomy was performed due to BSI of 15. The resected lobe was sent to a pathologist for histopathological analysis. An impacted 1.3 cm non-surgical plastic foreign body was discovered in the right lower bronchus by the pathologist during the gross examination of the specimen (Figures 2, 3). The histology of the airway and lung parenchyma surrounding the foreign body showed chronic inflammation and reactive benign tissue growth. Post-operative recovery in the hospital lasted four days and was uneventful. The patient had no recollection of aspiration or choking in the past which could explain the foreign body, nor did she have risk factors for aspiration such as dementia, alcoholism, drug use, stroke, medications, etc. Subsequently, the patient has had no recurrence of infections in the one-year follow-up.

## Discussion

We have presented a case of non-cystic fibrosis bronchiectasis in a middle-aged woman due to retained foreign body which was not apparent with history, imaging or bronchoscopic findings. This cause of bronchiectasis in adults has been reported in the past but is rare. Prevalence of the non-cytic fibrosis bronchiectasis in adults was estimated to be between 340,000 and 522,000 patients in the United States alone in 2013 with women constituting about 67% of the disease population [4]. Pathophysiology of the disease includes infection and impaired drainage or impaired host defence system like in our patient. The ensuing host response system of mostly neutrophils, cytokines, reactive oxygen species and proteases causes chronic inflammation of the airways with subsequent ulceration and airway dilation leading to bronchiectasis [5].

Most of the acquired non-cystic fibrosis bronchiectasis (NCFB) is either due to known immune deficiencies, alpha-one antitrypsin deficiency, ABPA or chronic foreign body retention. The most common site for the lodgement of foreign body occurs in the right main bronchus since it is wider and more vertical compared to the left main bronchus making it the path of least resistance for the aspirated material [6]. Such patients usually present with episodes of choking, coughing or periodic wheezing and should raise the suspicion of aspirated foreign body. In adults, such episodes are usually associated with altered mental status [7].

The suspicion of foreign body aspiration in our patient was low due to the fact that it was not apparent on the CT of the chest in addition to the history. The foreign body of 1.3 cm was although large enough to be detected on CT [8], was made of radiolucent material and hence was difficult to detect. The bronchoscopic examination did not reveal the foreign body because of reactive tissue growth around it found to be so during pathological examination [9]. Based on our literature review, we did not encounter similar cases to the one we present here.

## Conclusions

Bronchiectasis as a consequence of foreign body retention is a well-known, possibly under-diagnosed entity. Although assumed to be easy to diagnose with history, it is far more complicated due to the absence of remembrance of aspiration and/or chocking by the patient. Pneumonia, atelectasis and bronchiectasis are some of the serious complications of foreign body aspiration with bronchiectasis as a consequence of longer periods of retention. Radio-opaque foreign-bodies are logically easier to detect radiographically than radio-lucent materials.

Diagnosis of the foreign body retention in our patient was difficult due to multitude of reasons including absence of history of aspiration, radio-lucency nature of the material and abatement of the material by chronic inflammatory tissue eluding diagnosis via computed tomography and bronchoscopy. It is imperative that the above entity be suspected in localized bronchiectasis even at the absence of history suggesting as such.

## References

[1] Lonni S, Chalmers JD, Goeminne PC (2015). Etiology of non-cystic fibrosis bronchiectasis in adults and its correlation to disease severity. Ann Am Thorac Soc.

[2] Chalmers JD, Smith MP, McHugh BJ, Doherty C, Govan JR, Hill AT (2012). Short- and long-term antibiotic treatment reduces airway and systemic inflammation in non-cystic fibrosis bronchiectasis. Am J Respir Crit Care Med.

[3] Chalmers JD, Goeminne P, Aliberti S (2014). The bronchiectasis severity index. An international derivation and validation study. Am J Respir Crit Care Med.

[4] Weycker D, Hansen GL, Seifer FD (2017). Prevalence and incidence of noncystic fibrosis bronchiectasis among US adults in 2013. Chron Respir Dis.

[5] King PT (2009). The pathophysiology of bronchiectasis. Int J Chron Obstruct Pulmon Dis.

[6] Baharloo F, Veyckemans F, Francis C, Biettlot MP, Rodenstein DO (1999). Tracheobronchial foreign bodies. CHEST J.

[7] James P, Christopher DJ, Balamugesh T, Thomas R, Gupta R (2006). Multiple foreign body aspiration and bronchiectasis. J Bronchology Interv Pulmonol.

[8] Pitiot V, Grall M, Ploin D, Truy E, Khalfallah SA (2017). The use of CT-scan in foreign body aspiration in children: a 6 years' experience. Int J Pediatr Otorhinolaryngol.

[9] Dikensoy O, Usalan C, Filiz A (2002). Foreign body aspiration: clinical utility of flexible bronchoscopy. Postgrad Med J.

